# Management of Children

**Published:** 1886-03

**Authors:** 


### CARE AND MANAGEMENT OF CHILDREN.

Only those who watch infants with intellingent discrimination know how often they suffer from fever. With this fever comes *thirst*- What does the mother put into that little dry mouth? Often nothing but milk! When we adults have fever do we find that milk relieves the thirst? Does it not rather increase it? Be assured, it is the same with the baby. With the slightest symptoms of fever, cold water administered with a teaspoon is the prescription of wisdom and mercy.

Mothers, do you know that when your babies are feverish, restless, and sleepless, you have at hand the means to give them relief and refreshing sleep? I do not mean opiates, for in the end *they* add to the fever. I refer to the warm bath. For babies it is a blessed institution. Better than all medicines, it will impart relief and restoration to the feverish and restless little folks. The warm bath is not appreciated. In addition to its charming effect upon the general conditions to which I have alluded, it is well to add, there is scarcely a local trouble of a temporary nature, as, for example, pain in the stomach or bowels, which will not give way upon immersing the body in the warm bath. The degree of temperature may be determined by the urgency of the symptoms. The greater the suffering the warmer should be the water, especially, if the patient be one of strong constitution. When the little sufferer becomes quiet or the skin moist, it should be taken out rubbed with soft, warm towels, and wrapped in a fresh warm blanket.

During the last five years of my professional management of the sick, I was in the habit of constantly resorting to the warm bath as above advised and always with the most satisfactory results. No other simple means in the treatment of sick children can be compared with it. In teething, the brain irritation and bowel affections are more relieved by a judicious use of the warm bath than by all other means.—*Babyhood.*

**A Chicago** doctor was about to anæsthetize a patient, when in answer to a question, he informed the victim that he would be entirely unconsious, and know nothing, until the offending growth had been removed. The patient accordingly commenced to fish his loose change out of his pocket. "Oh, you need not mind the fee until I am through," remarked the considerate doctor. "I don't intend to pay you yet; returned the patient, "I wish merely to count my money to see how much I have."—*Medical and Surgical Reporter.*

